# Supplementary material for: The Budapest Amyloid Predictor and Its Applications
Source: Biomolecules. 2021 Mar 26;11(4):500. doi: 10.3390/biom11040500 (PMC8067080; doi:10.3390/biom11040500)
Supplement: Supplementary file 1 [file biomolecules-11-00500-s001.pdf]

The Budapest Amyloid Predictor and its Applications Supporting Material

László Keresztes[a,**], Evelin Szögi[a,**], Bálint Varga[a], Viktor Farkas[c], András Perczel[c,d], Vince Grolmusz[a,b,*]

[a] *PIT Bioinformatics Group, Eötvös University, H-1117 Budapest, Hungary*
[b] *Uratim Ltd., H-1118 Budapest, Hungary*
[c] *MTA-ELTE Protein Modeling Research Group, H-1117 Budapest, Hungary*
[d] *Laboratory of Structural Chemistry and Biology, Eötvös University, H-1117, Budapest, Hungary*

The Precision Recall Curve

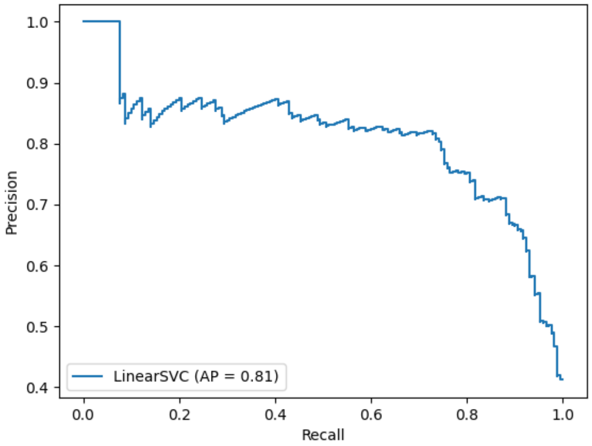

Figure S1: The precision-recall curve of the Budapest Amyloid Predictor. The area under the curve 0.8079. The average precision-recall score is 0.81

Ten-Fold Cross Validation

The Budapest Amyloid Predictor uses a fixed SVM: this SVM was trained on 948 hexapeptides, and tested on 467 hexapeptides from the Waltz dataset

*Corresponding author
**Joint first authors

(see the main text for details).

Here we describe a ten-fold cross validation of the SVM construction. Let us note that in each of the 10 runs we constructed a different SVM from different training- and test-sets. Therefore, this validation shows the power of the SVM construction in general, and not the power of the fixed SVM in the Budapest Amyloid Predictor in particular.

We also note that the Budapest Amyloid Predictor cannot use any consensus or averaging strategy from these ten-fold cross-validations, since then the main advantage of the predictor, which we have described in the Table 2 in the main text, would disappear.

In the ten-fold cross validation we used the following strategy: the Waltz dataset was partitioned into 10 pairwise distinct set, denoted by $W_1, W_2, \ldots, W_{10}$, such that they all contain the same number of hexapeptides and the same number of negative and positive examples of amyloidogenic hexapeptides, with a ± 1 margin. Let $W = \bigcup W_i$.

Then in turn i we applied $W - W_i$ as training-set and W_i as test set.

Table S1: the results of the 10-fold cross-validations

```
Train acc.: 0.8279654359780048
Round 1: 122 correct out of 142 tests.
Acc: 0.859155 TP: 43 TN: 79 FP: 12 FN: 8

Train acc.: 0.8554595443833464
Round 2: 105 correct out of 142 tests.
Acc: 0.739437 TP: 37 TN: 68 FP: 22 FN: 15

Train acc.: 0.826394344069128
Round 3: 117 correct out of 142 tests.
Acc: 0.823944 TP: 37 TN: 80 FP: 10 FN: 15

Train acc.: 0.826394344069128
Round 4: 120 correct out of 142 tests.
Acc: 0.845070 TP: 41 TN: 79 FP: 11 FN: 11

Train acc.: 0.819324430479183
Round 5: 123 correct out of 142 tests.
Acc: 0.866197 TP: 39 TN: 84 FP: 6 FN: 13

Train acc.: 0.8383045525902669
Round 6: 111 correct out of 141 tests.
Acc: 0.787234 TP: 39 TN: 72 FP: 18 FN: 12

Train acc.: 0.826530612244898
Round 7: 113 correct out of 141 tests.
Acc: 0.801418 TP: 36 TN: 77 FP: 13 FN: 15

Train acc.: 0.8281004709576139
Round 8: 113 correct out of 141 tests.
Acc: 0.801418 TP: 40 TN: 73 FP: 17 FN: 11

Train acc.: 0.8383045525902669
Round 9: 107 correct out of 141 tests.
Acc: 0.758865 TP: 38 TN: 69 FP: 21 FN: 13

Train acc.: 0.8288854003139717
Round 10: 113 correct out of 141 tests.
Acc: 0.801418 TP: 35 TN: 78 FP: 12 FN: 16
```

Table S2: the analogue of Table 1 in the main text for cross validation Round 1

	1	2	3	4	5	6
A	-0.439110	-0.476725	-0.390776	-0.087359	-0.519131	-0.293811
R	-0.622902	-0.712244	-0.608506	-0.335147	-0.797482	-0.575953
N	-0.460280	-0.469784	-0.521713	-0.155615	-0.569755	-0.379696
D	-0.635288	-0.685677	-0.647739	-0.446603	-0.788876	-0.500742
C	-0.058633	-0.315037	0.112366	0.021324	-0.154724	-0.024603
Q	-0.471809	-0.466079	-0.334978	-0.364218	-0.581345	-0.450002
E	-0.753920	-0.642889	-0.444872	-0.218974	-0.787215	-0.551301
G	-0.336540	-0.668023	-0.508523	-0.463541	-0.389835	-0.461230
H	-0.370771	-0.382870	-0.237349	-0.301302	-0.413232	-0.356055
I	-0.167334	-0.246692	0.289931	0.163871	-0.153839	-0.200579
L	-0.235565	-0.354866	-0.028373	0.169587	-0.300284	-0.207400
K	-0.501858	-0.723673	-0.602139	-0.313212	-0.796218	-0.430012
M	-0.283565	-0.496353	-0.035486	-0.068176	-0.186568	-0.268206
F	-0.285304	-0.316615	0.079576	0.021418	-0.213189	-0.191122
P	-0.938315	-0.790621	-0.725810	-0.735360	-0.602195	-0.723773
S	-0.471283	-0.543877	-0.438109	-0.311459	-0.650414	-0.348269
T	-0.441386	-0.573924	-0.313991	-0.216417	-0.699318	-0.318488
W	-0.322773	-0.499182	-0.179555	0.043445	-0.129724	-0.333561
Y	-0.337889	-0.376835	-0.174386	-0.055217	-0.335818	-0.172188
V	-0.090288	-0.332422	0.238884	0.218496	-0.339191	-0.013349

Table S3: the analogue of Table 1 in the main text for cross validation Round 2

	1	2	3	4	5	6
A	-1.078071	-0.700593	-0.617813	-0.007913	-0.928112	-0.474279
R	-1.412506	-2.186083	-1.786596	-0.418194	-1.050713	-1.022701
N	-0.992760	-0.764469	-0.544370	0.093936	-0.861888	-0.644175
D	-1.338563	-1.091058	-1.007986	-0.992505	-1.302572	-0.731304
C	-0.136922	-0.429890	0.242296	0.347428	-0.210918	0.186396
Q	-0.959700	-0.775441	-0.181715	-0.488807	-0.869635	-0.708054
E	-1.708835	-0.965551	-0.485424	-0.039619	-1.107309	-0.685536
G	-0.925022	-0.958521	-0.565213	-0.483144	-0.585163	-0.768921
H	-0.986854	-0.518175	0.223736	-0.708369	-0.509160	-0.634557
I	-0.707622	-0.455941	0.459833	0.270525	-0.319831	-0.772537
L	-0.823033	-0.510744	-0.157294	0.357740	-0.626398	-0.325869
K	-1.076932	-1.224313	-0.773038	-0.194076	-1.179887	-0.717077
M	-1.010752	-1.123637	-0.144632	0.107561	-0.167200	-0.568633
F	-0.825158	-0.494909	0.068624	0.084333	-0.373686	-0.424606
P	-2.511490	-1.463690	-1.533390	-1.529404	-0.944620	-1.485418
S	-1.147527	-0.811767	-0.288878	-0.337947	-1.025958	-0.639296
T	-1.088200	-0.927320	-0.440994	0.013200	-1.064173	-0.553839
W	-0.938884	-1.304571	-0.277253	0.413988	-0.350874	-0.856913
Y	-1.031414	-0.681919	-0.336609	0.109732	-0.566858	-0.120150
V	-0.623318	-0.704902	0.316567	0.394521	-0.564800	-0.037814

Table S4: the analogue of Table 1 in the main text for cross validation Round 3

	1	2	3	4	5	6
A	-0.407095	-0.442474	-0.424331	-0.126625	-0.499006	-0.357778
R	-0.647278	-0.736189	-0.628376	-0.436709	-0.721311	-0.532222
N	-0.424088	-0.482276	-0.512701	-0.225516	-0.516196	-0.445536
D	-0.602844	-0.684338	-0.662273	-0.499889	-0.735750	-0.567666
C	-0.029203	-0.255373	0.096754	0.042693	-0.163510	-0.059181
Q	-0.469061	-0.466997	-0.358209	-0.451596	-0.555929	-0.519779
E	-0.726901	-0.695087	-0.507284	-0.262504	-0.756173	-0.598856
G	-0.312561	-0.595509	-0.530825	-0.458093	-0.314193	-0.475550
H	-0.384507	-0.410268	-0.264588	-0.403488	-0.379091	-0.394069
I	-0.183557	-0.235400	0.315716	0.083992	-0.020566	-0.270723
L	-0.188470	-0.293456	-0.049597	0.053146	-0.197227	-0.282211
K	-0.491963	-0.710608	-0.611144	-0.346326	-0.734402	-0.478574
M	-0.275056	-0.446084	-0.044201	-0.122378	-0.146290	-0.372187
F	-0.246260	-0.252492	0.045415	-0.049888	-0.061521	-0.273514
P	-0.827301	-0.754213	-0.763565	-0.829708	-0.498015	-0.806149
S	-0.449539	-0.491640	-0.458372	-0.340425	-0.604036	-0.366865
T	-0.437504	-0.513494	-0.313608	-0.245185	-0.631908	-0.373990
W	-0.298824	-0.437829	-0.089664	0.011575	-0.063729	-0.429275
Y	-0.316853	-0.312041	-0.199782	-0.131938	-0.248073	-0.220958
V	-0.090474	-0.271982	0.208385	0.141568	-0.230544	-0.042006

Table S5: the analogue of Table 1 in the main text for cross validation Round 4

	1	2	3	4	5	6
A	-0.412488	-0.519148	-0.363124	-0.116835	-0.454720	-0.277723
R	-0.666344	-0.813422	-0.575336	-0.321848	-0.705104	-0.455845
N	-0.423042	-0.541050	-0.497358	-0.181278	-0.492916	-0.320072
D	-0.667236	-0.732177	-0.664629	-0.450458	-0.727028	-0.412854
C	-0.054017	-0.260436	0.109161	0.028811	-0.239601	0.050607
Q	-0.494460	-0.507272	-0.327513	-0.390669	-0.536184	-0.359221
E	-0.767139	-0.730459	-0.491329	-0.165175	-0.723519	-0.489629
G	-0.271413	-0.706657	-0.539443	-0.497089	-0.278502	-0.345586
H	-0.459906	-0.487099	-0.198040	-0.316385	-0.322955	-0.355444
I	-0.181585	-0.287651	0.253991	0.144853	-0.006674	-0.127165
L	-0.234899	-0.389044	-0.008528	0.123138	-0.151745	-0.142598
K	-0.470211	-0.823006	-0.553868	-0.333572	-0.683289	-0.332392
M	-0.296152	-0.542082	0.001253	-0.065191	-0.114514	-0.236960
F	-0.287897	-0.397458	0.047663	0.008071	-0.066353	-0.129829
P	-0.927785	-0.836527	-0.751850	-0.793459	-0.524418	-0.663936
S	-0.458870	-0.579081	-0.385546	-0.317539	-0.588862	-0.252033
T	-0.478858	-0.595424	-0.266849	-0.208575	-0.613034	-0.254722
W	-0.312059	-0.527248	-0.143728	0.064409	-0.036329	-0.237720
Y	-0.338126	-0.472563	-0.235016	-0.062994	-0.218710	-0.026096
V	-0.106687	-0.350360	0.216127	0.209117	-0.223417	0.066043

Table S6: the analogue of Table 1 in the main text for cross validation Round 5

	1	2	3	4	5	6
A	-0.480383	-0.372494	-0.439925	-0.117238	-0.531735	-0.257909
R	-0.667236	-0.647384	-0.565130	-0.361910	-0.790268	-0.548182
N	-0.458445	-0.412073	-0.525190	-0.167107	-0.579181	-0.398351
D	-0.653956	-0.631402	-0.663203	-0.416002	-0.827980	-0.536788
C	-0.088888	-0.251423	0.081127	0.061649	-0.222860	-0.064124
Q	-0.501958	-0.417372	-0.363262	-0.385531	-0.599443	-0.436062
E	-0.780159	-0.597687	-0.536872	-0.224574	-0.822811	-0.533237
G	-0.330852	-0.551749	-0.547587	-0.441712	-0.331651	-0.431709
H	-0.420470	-0.348361	-0.229963	-0.330436	-0.441034	-0.389504
I	-0.226504	-0.136100	0.309112	0.089064	-0.127408	-0.214147
L	-0.297132	-0.180833	-0.053484	0.103047	-0.268632	-0.191573
K	-0.531887	-0.631413	-0.601717	-0.329420	-0.833466	-0.426716
M	-0.362155	-0.390996	-0.022395	-0.083105	-0.213290	-0.302079
F	-0.359253	-0.197379	0.057886	-0.010597	-0.154183	-0.213559
P	-0.969524	-0.614510	-0.765145	-0.738396	-0.471498	-0.710551
S	-0.510170	-0.451512	-0.456977	-0.297023	-0.645448	-0.318552
T	-0.519897	-0.448242	-0.289990	-0.202026	-0.660715	-0.293998
W	-0.355604	-0.328556	-0.132242	0.026202	-0.116969	-0.350032
Y	-0.375820	-0.276252	-0.153490	-0.078140	-0.282820	-0.161495
V	-0.139404	-0.220263	0.220478	0.193894	-0.326373	0.005058

Table S7: the analogue of Table 1 in the main text for cross validation Round 6

	1	2	3	4	5	6
A	-0.444277	-0.402136	-0.400983	-0.213132	-0.560068	-0.302560
R	-0.605337	-0.672125	-0.600534	-0.424077	-0.848539	-0.517102
N	-0.445486	-0.388948	-0.505184	-0.240689	-0.642935	-0.412096
D	-0.684142	-0.567615	-0.669916	-0.549283	-0.870431	-0.472867
C	-0.079192	-0.142575	0.084924	0.039937	-0.256171	-0.019408
Q	-0.471652	-0.374841	-0.352212	-0.442264	-0.637358	-0.430393
E	-0.801735	-0.590440	-0.538626	-0.312359	-0.872842	-0.549106
G	-0.310869	-0.538892	-0.560049	-0.562960	-0.435769	-0.452675
H	-0.353145	-0.359264	-0.297433	-0.385214	-0.400840	-0.312756
I	-0.144972	-0.136767	0.282338	0.061875	-0.171093	-0.194561
L	-0.191402	-0.182176	-0.052197	0.030632	-0.315582	-0.187719
K	-0.452187	-0.672201	-0.644190	-0.385408	-0.835145	-0.415421
M	-0.243033	-0.424858	-0.056888	-0.124469	-0.216758	-0.249094
F	-0.280202	-0.209321	0.106156	-0.028497	-0.214629	-0.140620
P	-0.907555	-0.657527	-0.729946	-0.828161	-0.658220	-0.691764
S	-0.512486	-0.386211	-0.394595	-0.428218	-0.726061	-0.325379
T	-0.496696	-0.418521	-0.275873	-0.306355	-0.760976	-0.328834
W	-0.307484	-0.404753	-0.134260	0.028816	-0.158414	-0.291021
Y	-0.351599	-0.343900	-0.129266	-0.134219	-0.339267	-0.128631
V	-0.096394	-0.201490	0.250910	0.121014	-0.328715	-0.017054

Table S8: the analogue of Table 1 in the main text for cross validation Round 7

	1	2	3	4	5	6
A	-0.423072	-0.322525	-0.436258	-0.154027	-0.528924	-0.427972
R	-0.619588	-0.632892	-0.656948	-0.371419	-0.741094	-0.543496
N	-0.461176	-0.393165	-0.554435	-0.184343	-0.554920	-0.496127
D	-0.634400	-0.531432	-0.740112	-0.517252	-0.738400	-0.619322
C	-0.048013	-0.155814	0.088222	0.015518	-0.221948	-0.123597
Q	-0.433966	-0.378761	-0.381176	-0.390899	-0.602564	-0.486497
E	-0.735810	-0.476835	-0.559616	-0.323037	-0.764239	-0.660383
G	-0.378576	-0.604691	-0.602013	-0.460746	-0.324581	-0.565315
H	-0.348453	-0.320536	-0.248517	-0.378156	-0.433979	-0.440109
I	-0.177897	-0.138851	0.273069	0.115440	-0.109059	-0.331242
L	-0.193673	-0.170395	-0.073933	0.108798	-0.307616	-0.401192
K	-0.482638	-0.628583	-0.688569	-0.342913	-0.746579	-0.503224
M	-0.274658	-0.368791	-0.032009	-0.118907	-0.219799	-0.425243
F	-0.272839	-0.188356	0.068785	0.015013	-0.130500	-0.336631
P	-0.879885	-0.667691	-0.756451	-0.803967	-0.541438	-0.854324
S	-0.475437	-0.364307	-0.445022	-0.331169	-0.640471	-0.425939
T	-0.464013	-0.401554	-0.320685	-0.239584	-0.675040	-0.399225
W	-0.328771	-0.354696	-0.168641	0.007106	-0.091967	-0.444440
Y	-0.318874	-0.267330	-0.240674	-0.052959	-0.275260	-0.243899
V	-0.104776	-0.194436	0.217162	0.167335	-0.291862	-0.095654

Table S9: the analogue of Table 1 in the main text for cross validation Round 8

	1	2	3	4	5	6
A	-0.480447	-0.365924	-0.419787	-0.140594	-0.501793	-0.376547
R	-0.647062	-0.610035	-0.640979	-0.361163	-0.755629	-0.587364
N	-0.461734	-0.425179	-0.547905	-0.195907	-0.568608	-0.491098
D	-0.654737	-0.551139	-0.708577	-0.477220	-0.769745	-0.636876
C	-0.124074	-0.173685	0.132230	0.068417	-0.196425	-0.059640
Q	-0.474831	-0.388898	-0.419410	-0.368442	-0.572704	-0.515604
E	-0.770948	-0.528690	-0.626890	-0.248304	-0.758657	-0.687422
G	-0.375577	-0.562986	-0.601010	-0.452909	-0.306711	-0.494537
H	-0.377765	-0.336032	-0.227156	-0.360535	-0.402490	-0.390174
I	-0.204168	-0.096768	0.313388	0.140885	-0.066979	-0.312950
L	-0.275191	-0.165664	-0.028192	0.094130	-0.203878	-0.296498
K	-0.500484	-0.647017	-0.628950	-0.336419	-0.762583	-0.536528
M	-0.312326	-0.401973	-0.001863	-0.060721	-0.160210	-0.310866
F	-0.295792	-0.179755	0.085370	0.003625	-0.058705	-0.321371
P	-0.983756	-0.711640	-0.777157	-0.721617	-0.550714	-0.836105
S	-0.540867	-0.387923	-0.430216	-0.322074	-0.642339	-0.378964
T	-0.548283	-0.444041	-0.308765	-0.232900	-0.675777	-0.356067
W	-0.278976	-0.367575	-0.095092	0.022129	-0.057302	-0.388765
Y	-0.361565	-0.248956	-0.157097	-0.090223	-0.259740	-0.204396
V	-0.163557	-0.166234	0.257990	0.218238	-0.288820	-0.064514

Table S10: the analogue of Table 1 in the main text for cross validation Round 9

	1	2	3	4	5	6
A	-0.384853	-0.438164	-0.402362	-0.099824	-0.580226	-0.363684
R	-0.598078	-0.763811	-0.603210	-0.382736	-0.792363	-0.510010
N	-0.393817	-0.480006	-0.526159	-0.183739	-0.577845	-0.470692
D	-0.563846	-0.688418	-0.731217	-0.443750	-0.844476	-0.656925
C	-0.057921	-0.213426	0.032444	0.079619	-0.245093	-0.181877
Q	-0.431080	-0.467783	-0.355166	-0.379414	-0.642015	-0.496727
E	-0.627824	-0.685952	-0.546516	-0.222273	-0.866043	-0.635608
G	-0.325928	-0.556624	-0.525506	-0.479216	-0.383720	-0.554540
H	-0.334142	-0.470044	-0.221745	-0.382779	-0.399022	-0.385232
I	-0.160026	-0.245856	0.310696	0.137545	-0.172535	-0.290656
L	-0.177892	-0.281800	-0.000181	0.116389	-0.304700	-0.276128
K	-0.445775	-0.741344	-0.571212	-0.366811	-0.788037	-0.476466
M	-0.258248	-0.517349	-0.049681	-0.063477	-0.257676	-0.372513
F	-0.251667	-0.323720	0.129313	0.015698	-0.158585	-0.266144
P	-0.849163	-0.707119	-0.762313	-0.791428	-0.590813	-0.815657
S	-0.451863	-0.478420	-0.439364	-0.304422	-0.683134	-0.428782
T	-0.483236	-0.543945	-0.321794	-0.261527	-0.721361	-0.392034
W	-0.282281	-0.497833	-0.155185	0.065710	-0.144416	-0.430652
Y	-0.308001	-0.385166	-0.163636	-0.059732	-0.348115	-0.218312
V	-0.083623	-0.270749	0.241116	0.234612	-0.329799	-0.076290

Table S11: the analogue of Table 1 in the main text for cross validation Round 10

	1	2	3	4	5	6
A	-0.382844	-0.451756	-0.400909	-0.161194	-0.576072	-0.363942
R	-0.523118	-0.712738	-0.580775	-0.474429	-0.824620	-0.587733
N	-0.416325	-0.468599	-0.562353	-0.236627	-0.618061	-0.514586
D	-0.545854	-0.568988	-0.706035	-0.524670	-0.854180	-0.579422
C	-0.001978	-0.240435	0.073717	0.068688	-0.273213	-0.113576
Q	-0.415266	-0.426483	-0.417723	-0.439431	-0.665242	-0.485338
E	-0.640222	-0.584133	-0.559255	-0.319895	-0.898158	-0.614656
G	-0.235558	-0.572686	-0.536877	-0.548813	-0.378146	-0.534540
H	-0.329536	-0.373551	-0.242204	-0.353418	-0.475123	-0.421664
I	-0.090636	-0.177374	0.271707	0.105298	-0.160215	-0.294778
L	-0.130589	-0.277683	-0.038642	0.080344	-0.328302	-0.342504
K	-0.387809	-0.733808	-0.609762	-0.509618	-0.836113	-0.502748
M	-0.213721	-0.465571	-0.015180	-0.082687	-0.232402	-0.355560
F	-0.197904	-0.228554	0.048335	-0.029484	-0.203612	-0.294952
P	-0.834760	-0.684123	-0.800466	-0.794672	-0.681508	-0.834883
S	-0.440736	-0.474320	-0.464264	-0.320099	-0.751515	-0.435192
T	-0.443887	-0.485235	-0.339080	-0.242940	-0.754710	-0.400748
W	-0.197405	-0.371913	-0.090921	-0.012537	-0.136554	-0.424839
Y	-0.222870	-0.297468	-0.178655	-0.133863	-0.365709	-0.254011
V	-0.040421	-0.240315	0.210686	0.173225	-0.371702	-0.054475
